# Systemic Vascular Resistance and Myocardial Work Analysis in Hypertrophic Cardiomyopathy and Transthyretin Cardiac Amyloidosis with Preserved Left Ventricular Ejection Fraction

**DOI:** 10.3390/jcm13061671

**Published:** 2024-03-14

**Authors:** Cesare de Gregorio, Giancarlo Trimarchi, Denise Cristiana Faro, Cristina Poleggi, Lucio Teresi, Fabrizio De Gaetano, Concetta Zito, Francesca Lofrumento, Ioanna Koniari, Roberto Licordari, Nicholas G. Kounis, Ines Paola Monte, Gianluca Di Bella

**Affiliations:** 1 Department of Clinical and Experimental Medicine, University Hospital of Messina, 98125 Messina, Italy; giancarlo.trimarchi18@gmail.com (G.T.); cristinapoleggi@gmail.com (C.P.); lucioteresi@gmail.com (L.T.); concetta.zito@unime.it (C.Z.); franci.lofru@gmail.com (F.L.); robertolicordari@gmail.com (R.L.); gianluca.dibella@unime.it (G.D.B.); 2 Department of Surgery and Medical-Surgical Specialties, University of Catania, 95123 Catania, Italy; denisefaro88@gmail.com (D.C.F.); fabridegafp7@gmail.com (F.D.G.); inemonte@unict.it (I.P.M.); 3 Liverpool Centre for Cardiovascular Science, Liverpool L14 3PE, UK; iokoniari@yahoo.gr; 4 Department of Medicine, Division of Cardiology, University Hospital of Patras, 26504 Patras, Greece; ngkounis@otenet.gr

**Keywords:** cardiac amyloidosis, heart failure with preserved left ventricular ejection fraction, hypertrophic cardiomyopathy, systemic vascular resistance, strain echocardiography, ventricular arterial coupling

## Abstract

**Background**: The pathophysiological impact of systemic vascular resistance (SVR) and pressure–strain loop-derived global myocardial work index (GWI) in hypertrophic cardiomyopathy (HCM) and transthyretin cardiac amyloidosis (ATTR) has been randomly investigated. **Methods**: Both SVR and GWI were assessed in outpatients consecutively referred at two Italian cardiology departments for heart failure with preserved left ventricular ejection fraction (LVEF), affected by either nonobstructive HCM or wild-type ATTR. Based on relevant cross-tabulations, the patients were gathered into 4 functional classes according to cut-off values of 1440 dyne/s/cm^−5^ for SVR, and 1576 mm Hg% for GWI, as suggested by previous studies. **Results**: A total of 60 patients, 30 in each group, aged 61 ± 16 years, with 78% males, were studied. HCM patients were younger than those with ATTR and in a better clinical condition (23% HCM vs. 77% ATTR were NYHA class II-III, *p* < 0.001). Overall, 51 patients (85%) showed a high SVR, 21/30 HCM (70%), and 30 ATTR (100%) (*p* < 0.005). Both SVR and GWI (expressions of ventricular–arterial coupling) were impaired in 43% of HCM patients (showing greater LV concentric hypertrophy) and 93% of ATTR patients (in advanced NYHA functional class) (*p* < 0.001). **Conclusions**: A substantial percentage of present study population showed impaired SVR and/or GWI, despite preserved LVEF. The proposed classification may shed further light on the pathophysiological and clinical characteristics of such hypertrophic phenotypes.

## 1. Introduction

Although the concept of systemic vascular resistance (SVR) has long been recognized through experimental and human studies on hypertensive and cerebrovascular patients [1,2,3,4], only recently it gained popularity in chronic heart failure (HF) settings [5,6]. SVR can be calculated by ultrasound-derived parameters, noninvasively, and it is considered a marker of left ventricular (LV) afterload, aortic arterial elastance, and distal vascular resistance to blood flow [1,2,5,6]. Patients with chronic HF may demonstrate changes in SVR related to higher arteriolar and microvascular tone, sympathetic drive, hyperactivity of the renin–angiotensin–aldosterone system and/or blood viscosity [3,4,5,6,7]. Hemodynamic studies have provided a normal SVR range varying from 900 to 1440 dynes/s/cm^−5^ [8], while its calculation can contribute to differentiate underlying pathophysiologic mechanisms and guide therapy, even in the acute HF settings. In fact, hypotensive patients, due to sepsis, can reveal low SVR, while hypotension resulting from cardiogenic shock may be linked to elevated SVR [9]. From this perspective, the evaluation of SVR and LV function underlies the concept of ventricular–arterial coupling (VAC), the interplay between the heart and the arterial system, which is quite hard to be reproduced noninvasively. VAC offers the unique chance of analyzing the cardiovascular system adaptation to various clinical settings, including hypertrophic phenotypes, whose studies are lacking [10]. Recent European documents indicate that global longitudinal strain (GLS) by speckle-tracking echocardiography and pressure–strain loop-derived myocardial work (MW) analyses (especially global work index [GWI]) can be reliable surrogates of VAC [11].

At the present time, hypertrophic cardiomyopathy (HCM) and transthyretin cardiac amyloidosis (ATTR) are common hypertrophic phenotypes in clinical practice. We recently demonstrated that, despite similar echocardiographic presentation, MW parameters are more impaired in ATTR than in HCM patients, correlated to their reduced left ventricular ejection fraction (LVEF) [12,13]. The present study aimed to investigate whether the combined use of MW and SVR can be helpful to interpret these cardiovascular conditions in the same hypertrophic settings with preserved LVEF.

## 2. Materials and Methods

This was an observational study in consecutive adult patients admitted to the outpatient HF units at the cardiology departments of G. Martino University Hospital (Messina, Italy) and G. Rodolico University Hospital (Catania, Italy) from October 2022 to October 2023, who were scheduled to a clinical follow up or screening for HCM or ATTR.

Admission criteria were as follows: (a) age > 18 years; (b) HCM and wild-type ATTR according to current diagnostic criteria [14,15]; (c) good technical quality of the transthoracic echocardiogram; (d) preserved LV ejection fraction (>50% on two-dimensional imaging); and (e) sinus rhythm. Exclusion criteria were active (or previous) ischemic heart disease, systemic conditions with potential interference on cardiac function, severe heart valve disease, permanent atrial fibrillation, cancer, light chain (AL) amyloidosis, and mutated ATTR.

Careful patient screening was mandatory on enrollment through clinical history, physical examination, resting electrocardiogram (ECG), basic echocardiographic examination, advanced diagnostic techniques, and genetic testing, according to the current guidelines on cardiomyopathies [16]. Primary nonobstructive HCM was confirmed in patents presenting with all of the following criteria [14]: (1) LV wall thickness ≥ 15 mm in any myocardial wall segment, in a nondilated chamber and in the absence of relevant causes leading to LV hypertrophy; (2) typical ECG pattern; (3) family history of HCM, except in the case of an individual suspected to be the proband; (4) LV outflow tract gradient ≤ 30 mmHg at rest, exercise and/or Valsalva maneuver; and (5) genetic testing, if available.

Diagnoses of wild-type ATTR were made according to all of the following criteria [15]: (1) clinical history; (2) LV hypertrophy (wall thickness > 12 mm in any myocardial wall segment) on echocardiogram or cardiac magnetic resonance, checking for extra-ventricular features of ATTR as well (18); (3) discrepancy between ECG signs and echo criteria for LV hypertrophy; (4) genetic testing to rule out mutative forms; and (5) total body 99m Technetium–Pyrophosphate bone scintigraphy, showing a Perugini score 2 or 3.

The observational design was approved by the local Cardiology Research Board, ensuring the patient’s data privacy.

### 2.1. Transthoracic Echocardiography and Formulas

Ultrasound studies were all performed with the same vendor machine (Vivid E95; GE Vingmed Ultrasound, Horten, Norway). Conventional mono- and two-dimensional measurements, color-Doppler sampling and advanced analyses (GLS and MW) were performed in each patient and stored in a digital system, whereas post-processing analysis required a skilled examiner (physician). Transthoracic echocardiogram comprised imaging from the parasternal long-axis, short-axis, and 2-, 3-, and 4-chamber apical views, and the measurements were performed according to the current European/American guidelines [17].

As mentioned above, the hypertrophic phenotype was assessed by measuring the greatest wall thickness in the LV chamber. LV end-diastolic/systolic volumes were achieved by all apical views using the Simpson rule triplane method. Atrial chamber volumes were measured using the biplane method. Cardiac chamber volumes were then indexed to the body surface area (BSA). LV ejection fraction was measured automatically by the software, after checking for correctness of the border by the examiners. Diastolic function was assessed with a pulsed–wave Doppler at the mitral inflow (peak early velocity = E wave), divided by the early diastolic tissue velocity (e’ wave), and measured as the average value between the basal septum and the lateral mitral annulus (E/e’ ratio). Dynamic obstructive physiology was ruled out by measuring the LV outflow tract peak systolic gradient with color-Doppler-guided continuous-wave Doppler sampling under resting or/and Valsalva maneuver or stress condition. Speckle-tracking analysis was performed with a frame rate of 50–60 per second. Adjustments of the region of interest were manually applied by the two experienced examiners, and strain measurements taken from the apical 2- 3- and 4-chamber views of the LV. GLS and MW were calculated using digitally stored videoclips from each patient, using a dedicate analytic software (GE EchoPAC PC v204, General Electric, Horten, Norway). The analysis of MW required the LV pressure–strain loop digital construction starting from the measurement of systolic blood pressure (BP) in the sitting position by using an appropriate brachial cuff, just before ultrasound imaging acquisition, after the patient had rested for 15 min. MW indices were the average measurement of the respective 17-segment model analyses. Based on our recent study, we evaluated the two most significant MW markers, the global work index (GWI), which is consistent of the total work performed from the mitral valve closure until its opening, plus isovolumetric contraction and relaxation, and the global constructive work (GCW), which is the myocardial work performed during all of the LV shortening [13].

Total SVR was calculated using the original formula (SVRecho) by Stefadouros et al. [3], with the correction (SVRc) proposed to attain the value as close as possible to the invasive hemodynamic determination, as follows:

SVRc = [(mBP/CO) × 80 × 0.865] + 216 dyne/s/cm^−5^

where mBP was the mean blood pressure (mm Hg) calculated by the formula [(systolic BP-diastolic BP)/3] + diastolic BP, and CO (L/m) was the product of LV stroke volume (SV) × heart rate (bpm).

In order to better investigate the underlying cardiovascular status, the patient population was divided into 4 classes, according to cut-off values of 1440 dyne/s/cm^−5^ representing the normal limit for SVRc, as suggested by previous studies [3,8], and of 1576 mm Hg% for GWI index. This latter value constituted the lowest range value from 1827 healthy participants, median aged 45 years, in the recent Copenhagen City Heart Study [18]. Secondary clinical cut-off points were also considered in the scatter plot diagram.

### 2.2. Statistics

The Shapiro–Wilk test was applied to evaluate whether the data set was normally distributed or not. Continuous variables are expressed in the form of mean ± standard deviation (SD), if normally distributed; or median and interquartile range [IQR], if not. Categorical variables are represented by the absolute number and their respective percentages (%). Between-group and in-group differences of clinical and echocardiographic characteristics were compared through Student’s *t* test or Mann–Whitney U test, as appropriate. Categorical variables were analyzed by the χ^2^ test for the overall assessment, and by the Fisher’s exact test for pairwise group comparisons.

Correlation coefficients were determined for establishing the intra-observer agreement in 10 randomly selected patients to evaluate reproducibility. The null hypothesis was considered rejected for *p*-values < 0.05 at two-tailed significance level. Statistical analysis was conducted using SPSS (IBM SPSS Statistics) version 26.

## 3. Results

From an initial cohort of 87 patients referred to both clinical centers, 27 (31%) were excluded because of obstructive HCM (*n* = 8, 9%), familiar ATTR (*n* = 8, 9%), AL amyloid variant (*n* = 6, 7%), permanent atrial fibrillation (*n* = 3, 3%), and inadequate acoustic imaging for strain and myocardial work assessments (*n* = 2, 2%). A total of 60 patients, mean aged 61 ± 16 years, 30 in each group, with 47 males (78%), were finally enrolled. Demographic and clinical characteristics of the respective patients are displayed in Table 1.

There was no difference regarding the proportion of male patients between the groups, but those with HCM were younger than those with ATTR. An advanced New York Heart Association (NYHA) functional class was evident in this latter group, although none of the individuals were class IV. Accordingly, 77% of ATTR patients complained with mild to moderate dyspnea on effort, compared to 23% of HCM patients (*p* < 0.001). Office BP measurements were similar as well, facilitating an interpretation of the target markers of LV dysfunction, MW and SVR. ATTR patients were all receiving treatment with Tafamidis at the dose of 61 mg daily. Every patient provided a written consent for the echocardiographic study.

### 3.1. Conventional Echocardiography Findings

The main measurements are exposed in Table 2. There were no significant between-group differences regarding LV end-diastolic volume index, ventricular septum thickness, and LV mass index (weakly higher in ATTR patients). Although the LVEF was lower in this group, largely due to higher end-systolic volumes and ensuing lower CO, the participants fulfilled the HFpEF criteria, according to the current guidelines [19]. Both groups revealed the same degree of left atrial chamber enlargement, but ATTR patients demonstrated greater LV diastolic dysfunction and lower GLS values than HCM patients.

### 3.2. Systemic Vascular Resistance and Myocardial Work Indices

**Figure 1:**
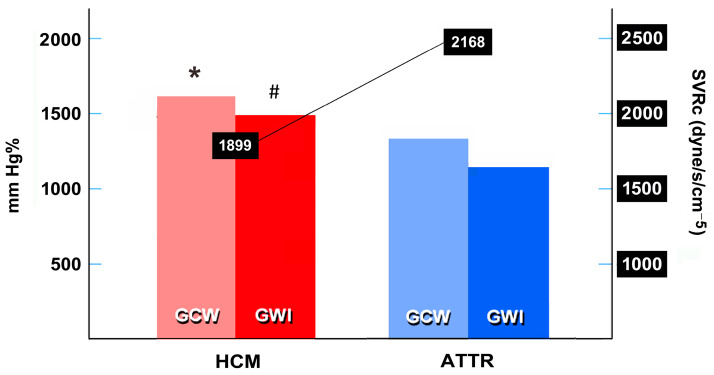
Median values of global myocardial constructive work (GCW), global work index (GWI) and systemic vascular resistance (SVRc) in the study population. ATTR, wild-type transthyretin amyloid cardiomyopathy HCM, hypertrophic cardiomyopathy; SVRc, systemic vascular resistance, corrected (see methods). HCM vs. ATTR: * *p* = 0.008; # *p* = 0.001. Differences in SVRc between the groups were significant (*p* = 0.008).

SVR_c_, GWI, and GCW were calculated in each patient (Table 2). Overall, 51 patients showed high SVR_c_ (85%), 21 were from the HCM (70%) group and 30 were from the ATTR group (100%) (*p* < 0.005). Moreover, SVR_c_ was reversely correlated to GWI and GCW (Figure 1). There was no linear correlation between mean blood pressure values and SVRc (Supplementary Materials). 

ATTR patients presented much lower GWI and GCW values than HCM patients, of which were consistent with our recent study results [13].

**Figure 2:**
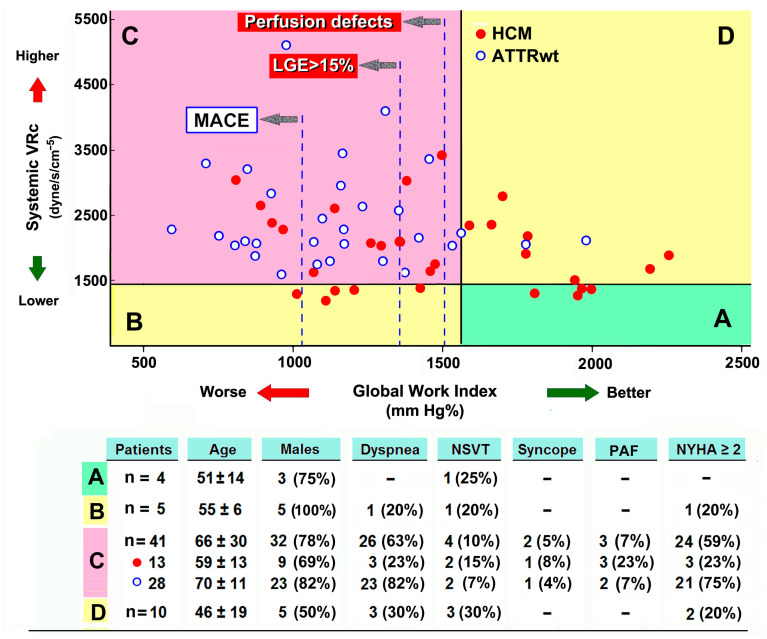
Scatter plot diagram showing four classes categorized according to SVR_c_ (cut-off 1440 dyne/cm/s^−5^) [3,8] and GWI (cut-off 1576 mm Hg%) [18]. Therefore, Class A included patients with low/normal SVRc and preserved GWI, then interpreted as the best functional class. Class B included patients with lower GWI and normal SVR_c_, taken as intermediate risk class. Class C included patients with higher SVR_c_ and impaired GWI, then considered as the worst one. Class D, another intermediate class due to high SVR_c_. Vertical dashed lines indicate the cut-off values by previous studies for impaired myocardial perfusion and late gadolinium enhancement (LGE) in HCM patients [21], and major cardiovascular events (MACE) in ATTR patients [22]. Tabulation below the graphics refers to some clinical findings from the patients belonging to different Classes (see also Table 3). ATTRwt, wild-type transthyretin cardiac amyloidosis; HCM, nonobstructive hypertrophic cardiomyopathy; NYHA, New York Heart Association functional class; NSVT, nonsustained ventricular tachycardia on 24-h ECG Holter monitoring; PAF, paroxysmal atrial fibrillation.

The novel functional classification was performed according to SVR_c_ and GWI value cross-tabulation (Figure 2, Table 3). Only nine patients out of the HCM group (15%) showed normal SVR_c_ (classes A or B), whereas 43% entered the poorest class C and 27% entered the intermediate class D. Except from the two cases, almost all ATTR patients (93%) were in class C.

Patients who had GWI < 1576 mm Hg% also included those with GCW < 1730 mm Hg%, which was suggested to be another prognostic factor in patients with nonobstructive HCM [20].

In Figure 2, three more cut-off values for GWI are represented in the diagram according to other studies. The value of GWI < 1517 mm Hg% was found in HCM patients with myocardial perfusion defects at stress-perfusion cardiac magnetic resonance, and the value of <1363 mm Hg% also discriminated the presence of late gadolinium enhancement > 15% of myocardial mass at cardiac magnetic resonance imaging [21]. The interrupted line indicating the risk of “MACE” at the cut-off value of GWI <1043 mm Hg% was previously found as an all-cause mortality risk factor in amyloid patients (61% wildtype ATTR) [22].

A paired ingroup HCM analysis demonstrated that patients in Class C had smaller LV chambers and higher septum thickness, as well as lower GLS than those in the other classes. Those were rather older, with a tendency towards lower LVEF and LAVi, but the subgroup consistency hindered reaching a statistical significance.

On the other hand, all ATTR patients showed higher SVR_c_ and only two out of these patients had normal GWI. Therefore, the large numerical discrepancy did not allow a between-class comparison (Table 3, bottom panel).

### 3.3. Intra-Observer Variability in MW and SVR Measurements

Intra-observer variability for the MW parameters and GLS was recently tested in our echo laboratories. Myocardial work index variability approximately was ±1.0% for GWI (bias 21 mm Hg%), ±4.5% for GCW (bias 62 mm Hg%), and 4.7% (bias −0.5%) for GLS. The variability of SVRc measurement was mainly related to an LV chamber volume assessment, then to the automated software recognition and operator adjustments, as required. Thus, inter- and intra-observed variability were in the order of ±5.5–6.0% (bias 0.2–0.3 L/min) for CO.

## 4. Discussion

Currently, the precise assessment of cardiovascular function in patients with cardiac hypertrophy is abridged by multitasking imaging technology that contributes to diagnosis and better management compared to previous years. Echocardiography and cardiac magnetic resonance remain the gold standards for the assessment of hypertrophic phenotypes at risk for HF [23,24].

The main findings from the present study indicate that the combined evaluation of SVRc and GWI can be useful to assign different functional (VAC) classes to HFpEF patients with either HCM or ATTR. For the first time to our knowledge, we disclosed SVR_c_ > 1440 dyne/m/sec^−5^ in 85% of the study population, but mostly in ATTR patients. Despite the lack of univocal cut-off values, an impairment in GWI resulted in 73% of patients, according to the lowest normal value reported by the CCHS study [18]. Taking these together, both study markers were impaired in 43% of HCM vs. 93% of ATTR patients, all classified in the poorest Class C. Of interest, the HCM individuals in Class C were quite older than in other classes, demonstrating a high degree of concentric LV hypertrophy, which usually constitutes a predictor of obstructive physiology [14].

According to Garcia Brás at al. [21], 18 patients of the HCM group (60%) also presented GWI values suggestive of impaired myocardial perfusion, and in 13 cases (43%), with possible late gadolinium enhancement > 15%.

However, only 13/18 (72%) and 8/13 patients (61%), respectively, also had higher SVRc; evidence that could further identify their risk for microvascular disease.

In amyloid patients, GWI < 1043 mm Hg% was linked to unfavorable outcomes in the study of Clemmersen et al. [22]. Although most ATTR patients in our series showed impaired GWI, only in 10 cases (33%) it was <1043 mm Hg%. Of note, in the ATTR population, SVR_c_ lost significance in discriminating the latter from other individuals.

The assessment of SVR_c_ is a long-standing methodological problem in cardiovascular diseases due to such a problematic reproduction of measurements across studies. However, early data by Wiggers in 1951 demonstrated that vascular reactivity, arterial elastance, and peripheral resistance do affect the LV function by imposing a rising afterload, at times, disproportionately to the basic cardiac condition [1].

Today, the cardiovascular interplay consists with the ultimate concept of ventricular-arterial coupling (VAC), which is a central target in various clinical and experimental studies [3,6,10,11]. The characterization of VAC should also include SVR, as the cardio-vascular functional interplay is the pivotal feature of systemic workload, also addressing potential therapeutic interventions in HF patients [9,11].

As suggested by Suga and Sagawa [25], and then confirmed by Sunagawa et al. [6], VAC belongs to the ratio of effective arterial elastance over end-systolic elastance. More recently, experts’ consensus documents suggested the VAC to be attained by advanced ultrasound-derived indices such as MW, and GWI has been proposed as a valid surrogate in various clinical settings [10,11,26]. By adopting the simple speckle-tracking modality over a single cardiac cycle, pressure–strain loop-derived MW computation likely outperforms the old methods for VAC. This is a breakthrough in the functional measurement as close as possible to traditional LV pressure–volume curves [11,18]. Accordingly, GWI and GCW were demonstrated to be prognostic factors in various clinical settings, including acute and chronic coronary syndromes, cardiomyopathies, and cardio-oncology [11,12,13,27,28]. Compared to conventional echocardiographic and Doppler techniques, the analysis of MW provides an amelioration in the study of VAC, also because imaging procession is load- and angle-independent. The use of a pressure–strain loop-derived analysis also allows a comprehensive assessment of the LV mechanics, with low inter- and intra-observer variability that ensures consistent results across different examiners.

However, further studies are still needed to better interpret the prognostic impact of MW indices and its qualified cut-off values in hypertrophic phenotypes [29]. A very low GWI value (<937 mm Hg%) was suggested as a predictor of cardiovascular mortality in 118 patients with cardiac amyloidosis, but such value suffered from low sensitivity and specificity [30].

Interestingly, Hiemstra et al. demonstrated a correlation between GCW and maximal LV wall thickness and diastolic function, suggesting the value < 1730 mm Hg% as a long-term outcome discriminator in HCM patients [20]. In our study, all HCM patients with GWI < 1576 mm Hg% also had GCW < 1730 mm Hg%, but only a proportion of them also showed high SVR_c_.

On the other hand, MW only in part reflects aortic compliance and SVR, which represent important factors of the CO and systemic perfusion. For these reasons, in the present study, we aimed at combining both GWI and SVR_c_ parameters to better interpret the cardiovascular function in such hypertrophic phenotypes. In non-obstructive HCM, adding SVR_c_ information to low GWI may indicate a subset of patients more inclined to microvascular and/or small coronary artery disease, both potential forecasters of tissue fibrosis even in the absence of epicardial vessel stenosis [21,31]. According to the clinical characteristics of Class C patients, the combined score of GWI and SVR might be more predictive of weak clinical conditions in ATTR patients, while linked to greater concentric hypertrophy in HCM patients, anticipating their potential obstructive physiology.

Even though all ATTR patients in this study were classified as HFpEF, the present results reinforce the pathophysiological theory that amyloidosis is a systemic illness in which the vascular compartment progressively deteriorates alongside the heart, somehow consistent with their clinical status. On the other hand, most ATTR patients present with autonomic dysfunction and BP variability, with a challenging vascular reactivity that affects their quality of life and prognosis [16,32,33]. This further confirms their cardiovascular system impairment is differently influenced by interstitial amyloid fibril accumulation than by genetic disorders featuring primary HCM [15,16,21,24,33,34]. Conversely, preserved MW indices and weakly higher (or normal) SVR_c_, as in two ATTR patients in our Class D, may suggest a less compromised systemic condition or early stage of disease, more likely to obtain benefit from specific therapy [23,24,28,32,35].

### Study Limitations

This study has important limitations. The number of patients was modest, due to the low incidence of ATTR among HF patients. Also, we needed to include only those with HFpEF, which is an even rarer condition in such clinical settings.

The method used for SVR computation by ultrasound may be imprecise, and no large studies have been conducted for the clinical validation of the formula proposed by Stefadouros et al. [3] in hypertrophic phenotypes. It should also be considered the role for the RA mean pressure in the native formula, although this likely was more helpful for pulmonary capillary resistance rather than SVR [10,12,35].

The calculation of MW needs multiple parameters and optimal acoustic window from all echocardiographic sections, which are not always excellent in such patients, and this may affect reproducibility [33]. Also, the fluctuation in BP measurements, especially in ATTR patients, hypertensives, or in the context of anxiety, can lead to relevant methodological errors and then impact the statistics and conclusions.

Also, we missed performing any comparison between SVRc, MW, and LV mass, because conventional echocardiographic methods likely fail to assess myocardial mass in a reliable way, compared with cardiac MRI. Four-dimensional echocardiography will surely address a better recognition of such interplay in both clinical settings. 

Finally, we were unable to assess the correlations between tested markers and the history of cardiovascular events, as well as tissue information from cardiac magnetic resonance imaging, because those findings were unattainable for some patients.

## 5. Conclusions

Impaired SVR_c_ and GWI were recognized in a significant proportion of the present study population, despite preserved LVEF. These markers are expressions of detrimental VAC that affects a sizable majority of ATTR (in advanced NYHA class) and over half of those with HCM (showing greater LV concentric hypertrophy).

The suggested classification may shed further light on different patient subsets and facilitate physicians to interpret their composite pathophysiological and clinical characteristics. Larger studies are required to validate the present results and establish any potential prognostic impact.

## Figures and Tables

**Table 1:** Demographic and clinical characteristics of study population.

	HCM (*n* = 30)	ATTR (*n* = 30)	*p* Value
Age, years	58 [54–73]	69 [62–78]	<0.001
Males	24 (80%)	23 (77%)	0.754
Body surface area, m^2.7^	1.9 [1.7–2.0]	1.7 [1.6–1.9]	<0.001
Systolic BP, mm Hg	128.8 ± 11.9	128.5 ± 12.7	0.925
Diastolic BP, mm Hg	77.4 ± 12.3	80.7 ± 7.9	0.226
Mean BP, mm Hg	94.5 ± 10.3	96.6 ± 7.4	0.375
Pulse pressure, mm Hg	51.4 ± 13.5	47.9 ±13.5	0.311
Heart rate, bpm	67.2 ± 11.3	68.4 ± 11.2	0.690
NYHA class I–III	1.4 ± 0.56	1.9 ± 0.46	<0.001
Dyspnea	7 (23%)	23 (77%)	<0.001
Family history of SD	7 (23%)	3 (10%)	0.166
Implantable cardioverter defibrillator	3 (10%)	1 (3%)	NA
Therapy			
Beta-blockers	25 (83%)	10 (33%)	<0.001
Calcium antagonists	11 (37%)	5 (17%)	0.080
ACE-inhibitors	8 (27%)	2 (7%)	0.07
ARB	1 (3%)	7 (23%)	0.023
Statins	6 (20%)	15 (50%)	0.015
Anti-coagulants	6 (20%)	6 (20%)	1.000
Anti-platelet drugs	5 (17%)	9 (30%)	0.222
Diuretics	4 (13%)	17 (57%)	<0.001
Tafamidis 65 mg	-	30 (100%)	NA

Values are numbers (%), mean ± SD, or median values [IQR], as appropriate. ACE, angiotensin-converting enzyme; ARB, angiotensin receptor antagonists; ATTR, wild-type transthyretin cardiac amyloidosis; BP, blood pressure; HCM, nonobstructive hypertrophic cardiomyopathy; NA, not available/performed; SCD, sudden cardiac death.

**Table 2:** Main echocardiographic measurements in both study groups.

	HCM (*n* = 30)	ATTR (*n* = 30)	*p* Value
LV end-diastolic volume index, mL/m^2.7^	51.4 [37.9–59.8]	49.7 [38.7–60.0]	0.831
LV end-systolic volume index, mL/m^2.7^	18.8 [15.0–25.9]	23.2 [17.2–27.1]	0.020
LV ejection fraction, %	63.4 [54.4–63.8]	55.0 [52.3–57.2]	<0.001
Stroke volume index, mL/m^2.7^	32.5 [23.2–36.3]	27.8 [21.5–31.8]	0.444
Cardiac output, L/min	3.9 [2.8–4.4]	3.4 [2.4–3.7]	0.004
Ventricular septum thickness, mm	15.2 [14.6–18.0]	15.5 [14.0–21.0]	0.688
Mitral E/tissue E’ velocity ratio	11.7 ± 5.0	16.8 ± 5.9	0.001
Global longitudinal strain, %	15.0 [16.0–10.3]	12.5 [14.5–10.0]	0.022
Global myocardial work index, mm Hg%	1468.5 ± 403.5	1149.7 ± 319.3	0.001
Global constructive work, mm Hg%	1442 [989–1554]	1141 [874–1356]	0.008
Left atrial volume index, mL/m^2.7^	43.7 ± 16.2	44.6 ± 15.5	0.991
SVR (corrected), dyne/s/cm^−5^	1899 [1693–2600]	2168 [2051–2860]	0.008

Measurements are expressed as mean values ± SD or median values [IQR], as appropriate. LV, left ventricle/ventricular; SVR, systemic vascular resistance.

**Table 3:** Classification of study population based on SVRc and GWI (see also Figure 2).

	HCM (*n* = 30)	ATTR (*n* = 30)	Total (*n* = 60)
Class A (Best class)	4 (13%)	-	4 (7%)
Class B (Impaired GWI)	5 (17%)	-	5 (8%)
Class C (Poorest class)	13 (43%)	28 (93%)	41 (68%)
Class D (Higher SVR_c_)	8 (27%)	2 (7%)	10 (17%)
Differential characteristics in subclass patients with HCM			
	**Class C (*n* = 13)**	**Other Classes (*n* = 17)**	***p*-Value**
Stroke volume index, mL/m^2.7^	26.7 ± 8.7	37.5 ± 10.8	0.005
LV end-diastolic volume index, mL/m^2.7^	43.7 ± 12.8	57.5 ± 16.1	0.015
GLS, %	13.0 ± 3.4	16.0 ± 4.3	0.043
IVS thickness, mm	18.2 ± 3.4	15.6 ± 2.8	0.029
LV ejection fraction, %	60.6 ± 6.1	64.8 ± 5.1	0.055
Age, years	58.7 ± 13.0	48.9 ± 16.2	0.076
Left atrial volume index, mL/m^2.7^	47.1 ± 20.8	41.1 ± 11.6	0.368

Measurements are expressed as mean values ± SD. HCM, hypertrophic cardiomyopathy; GLS, global longitudinal strain; IVS, interventricular septum; LV, left ventricle/ventricular.

## Data Availability

The data presented in this study are available on request from the corresponding author. The data underlying this article will be shared on reasonable request to the corresponding author.

## Supplementary Materials

The following supporting information can be downloaded at: https://www.mdpi.com/article/10.3390/jcm13061671/s1, Figure S1: Linear correlation between mean arterial BP and SVR in the whole study population.
