# Supplementary figures and images for: Systemic Vascular Resistance and Myocardial Work Analysis in Hypertrophic Cardiomyopathy and Transthyretin Cardiac Amyloidosis with Preserved Left Ventricular Ejection Fraction
Source: J Clin Med. 2024 Mar 14;13(6):1671. doi: 10.3390/jcm13061671 (PMC10970938; doi:10.3390/jcm13061671)

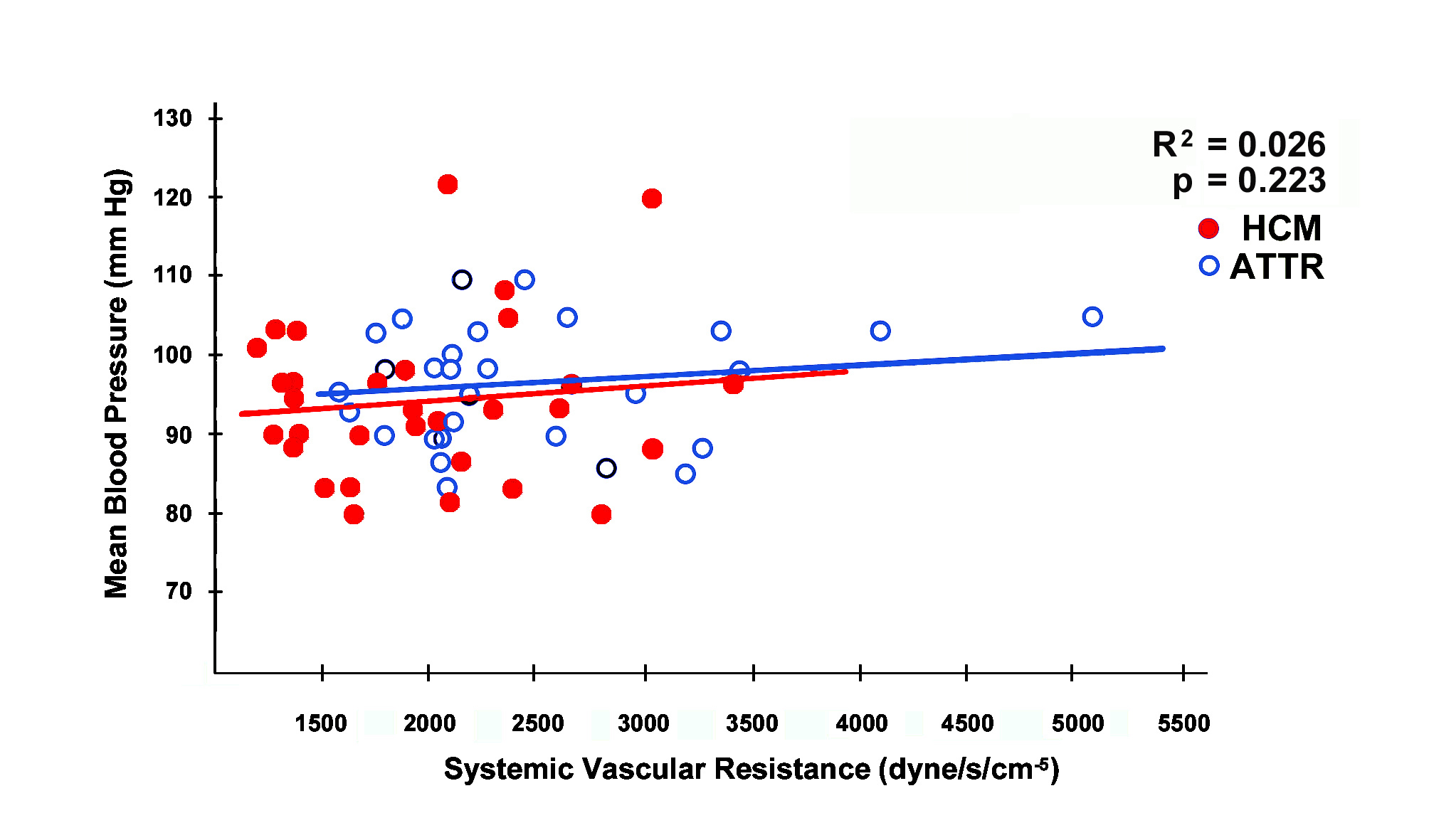

Supplement: Supplementary file 1 [file jcm-13-01671-s001.zip › jcm-2875932-supplementary.jpg]
